# Draft Genome Sequence of *Veillonella tobetsuensis* ATCC BAA-2400^T^ Isolated from Human Tongue Biofilm

**DOI:** 10.1128/genomeA.00808-15

**Published:** 2015-08-20

**Authors:** Izumi Mashima, Futoshi Nakazawa

**Affiliations:** a Japan Society for the Promotion of Science, Chiyoda-ku, Tokyo, Japan; b Department of Oral Microbiology, School of Dentistry, Health Sciences University of Hokkaido, Ishikari-Tobetsu, Hokkaido, Japan

## Abstract

Here, we report the draft genome sequence of *Veillonella tobetsuensis* ATCC-BAA 2400^T^. This bacterium has the remarkable ability to form oral biofilms. The genome is predicted to encode the necessary enzymes involved in the pathway that facilitates the conversion of lactate to propionate.

## GENOME ANNOUNCEMENT

The genus *Veillonella* consists of small, obligate anaerobic, Gram-negative cocci isolated from human oral cavities (1, 2). *Veillonella* species cannot catabolize sugars and instead rely on the fermentation of organic acids, such as lactate derived from *Streptococcus* spp., for the fermentation of sugars to propionic and acetic acids (3, 4). Additionally, *Veillonella* species demonstrate similar resistance to various antimicrobials, such as streptomycin, vancomycin, and tetracycline (5, 6), and were recently found to be resistant to penicillin and ampicillin (7).

*Veillonella tobetsuensis* was isolated from human tongue biofilm and established as a novel *Veillonella* species in 2013 (8). *V. tobetsuensis* has frequently been isolated from patients with periodontal diseases and in healthy individuals (9, 10). Additionally, it has the remarkable ability to form oral biofilms with *Streptococcus* species (11).

The draft genome of *V. tobetsuensis* ATCC BAA-2400^T^ was sequenced using Illumina HiSeq 2500 with sequencing runs for paired-end sequences. The bacterial DNA libraries were prepared using phenol-chloroform extraction and ethanol precipitation, as previously described (12). The genome was assembled into 49 contigs, ranging in size from 189 to 426,788 bp, using a sequence assembler for very short reads (Velvet version 1.2.08) and a *de novo* assembler designed to assemble high-throughput data (*Platanus* version 1.2.1) (13, 14). A synteny comparison with the genome was done using the closely related species *Veillonella parvula* DSM 2008 (accession no. NC_013520). Gene prediction was performed using Rapid Annotations using Subsystems Technology (RAST) (http://rast.nmpdr.org/) (15). The genes in the ATCC BAA-2400^T^ genome were assigned to Clusters of Orthologous Groups (COG) (16) categories, using BLASTp comparison with the COG database (http://www.ncbi.nlm.nih.gov/COG/), with an alignment *E* value cutoff of 10^-3^. Hokkaido System Science (Sapporo, Japan) performed the sequencing runs and read assembly libraries.

The draft genome sequence of ATCC BAA-2400^T^ was 2,161,277 bp, with a G+C content of 38.5% and 500-fold genome coverage. The genome sequence contained 1,913 coding sequences, 48 tRNAs, and 3 ribosomal RNAs. A large fraction of the protein-coding genes were assigned to functions in energy production and conversion and encompassed most of the genes known to be required for the conversion of lactate to propionate. This pathway was completely encoded in the ATCC BAA-2400^T^ genome and includes the characteristic methylmalonyl-coenzyme A (CoA) decarboxylase that generates a transmembrane electrochemical (Na^+^) gradient (17). This pathway is a critical component of the metabolic relationship of the oral cavity in which *Veillonella* is proposed to generate energy from the fermentation of lactate-producing bacteria, such as *Streptococcus* species (18). This system may largely contribute to the formation of biofilms with *Streptococcus* species (19).

### Nucleotide sequence accession numbers.

This draft genome sequence of *V. tobetsuensis* ATCC BAA-2400^T^ has been deposited at DDBJ/EMBL/GenBank under the accession numbers BBXI01000001 through BBXI01000049 (49 entries). The version described in this paper is the first version.
